# Comparative Efficacy of Medial Rectus Plication Versus Resection for Exotropia: A Systematic Review and Meta-Analysis

**DOI:** 10.7759/cureus.115460

**Published:** 2026-08-30

**Authors:** Mahmoud M Ismail, Aliaa Medhat Ahmed, Youssef Ali Youssef, Omar Alsharabi, Lamis Osama Taha, Mohamed Gamal Mohamed, Mohamed Khaled Mohamed, Asmaa Abdelbaset Elsayed Mohamed Elkasabi, Ebrahim Ali Mohammed Amrein, Mohamed Abdallah Alaswad, Ahmed Ayman Nomier, Mohammed Alaa Salem, Mohammed Almisbahy

**Affiliations:** 1 Pediatric Ophthalmology and Strabismus, King Abdullah Medical City, Makkah, SAU; 2 Ophthalmology, Suez Canal University, Ismailia, EGY; 3 College of Medicine, Suez Canal University, Ismailia, EGY; 4 College of Medicine, Mansoura University, Mansoura, EGY; 5 College of Medicine, Nahda University, Beni Suef, EGY; 6 College of Medicine, Suez University, Suez, EGY; 7 College of Medicine, Youcef El Khatib University of Health Sciences, Algiers, DZA; 8 College of Medicine, Zagazig University, Zagazig, EGY; 9 College of Medicine, Alexandria University, Alexandria, EGY; 10 College of Medicine, Emirates International University, Sanaa, YEM

**Keywords:** exotropia, medial rectus plication, medial rectus resection, meta-analysis, randomized control trial, strabismus surgery

## Abstract

Medial rectus resection has long been regarded as the standard muscle-strengthening procedure for the surgical management of exotropia. More recently, medial rectus plication has emerged as a vessel-sparing alternative that preserves the anterior ciliary circulation and may offer additional surgical advantages. However, the comparative efficacy of these two procedures remains uncertain. This systematic review and meta-analysis was conducted to compare the short-term clinical outcomes of medial rectus plication and medial rectus resection in patients undergoing surgery for exotropia. The review protocol was prospectively registered in the International Prospective Register of Systematic Reviews (PROSPERO) (#CRD420261326761). We performed a systematic search of electronic databases to identify randomized controlled trials directly comparing the two techniques. The primary outcomes were surgical success and postoperative change in deviation, while secondary outcomes included postoperative exodrift, operative time, surgical dose-response, and stereopsis. Six randomized controlled trials involving 352 patients were included, with 175 patients undergoing medial rectus plication and 177 patients undergoing medial rectus resection. No statistically significant difference was observed in surgical success (risk ratio: 0.86; 95% confidence interval: 0.66-1.14; p=0.22). Likewise, no significant difference was found in postoperative deviation change (mean difference 0.84 prism diopters, 95% confidence interval: -2.51 to 4.20; p=0.55). No statistically significant differences were observed between the two procedures with respect to postoperative exodrift, operative time, or stereopsis. Although a statistically significant difference was observed for surgical dose-response, its magnitude was small, and its clinical relevance remains uncertain. The certainty of evidence ranged from moderate to very low according to the Grading of Recommendations Assessment, Development and Evaluation (GRADE) approach. Overall, no statistically significant differences were identified between medial rectus plication and medial rectus resection across the evaluated short-term outcomes. Given its vessel-sparing nature and potential reversibility, medial rectus plication may represent a reasonable alternative to conventional medial rectus resection. Interpretation of these findings is limited by the small number of included trials, modest sample sizes, methodological limitations, and relatively short follow-up. Further large-scale, multicenter randomized controlled trials with longer follow-up are needed to confirm these findings and evaluate long-term surgical outcomes.

## Introduction and background

Strabismus surgery aims to restore ocular alignment. Plication, a muscle-tightening procedure, is considered less invasive than traditional resection, preserving the anterior ciliary vessels and offering potential procedural advantages such as reduced operative time and technical simplicity while theoretically reducing the risk of anterior segment ischemia through preservation of the anterior ciliary circulation [1,2]. However, the postoperative outcomes of plication versus resection remain controversial, with some studies reporting similar dose responses and others finding plication less predictable [3,4].

Exotropia (XT) is one of the most common forms of strabismus and is typically treated with monocular lateral rectus recession combined with medial rectus resection (R&R) or bilateral lateral rectus recession (BLRc). Comparative studies of medial rectus plication (MRP) and medial rectus resection (MRR) in these procedures have yielded conflicting results [5,6]. Although medial rectus resection remains the conventional strengthening procedure, medial rectus plication has gained increasing interest because it avoids muscle disinsertion and preserves anterior ciliary circulation. Whether these theoretical advantages translate into superior clinical outcomes remains uncertain.

Previous meta-analyses suffered from limitations including retrospective study designs, clinical heterogeneity, small sample sizes, and inadequate assessment of long-term outcomes [2,7]. Furthermore, recently published randomized controlled trials have provided additional evidence that warrants an updated quantitative synthesis [8-10].

## Review

This systematic review and meta-analysis compared the short-term surgical efficacy of medial rectus plication versus medial rectus resection in exotropia. The primary objectives were to evaluate and compare postoperative deviation change and surgical success. Surgical success was defined according to the criteria reported in the individual studies, with acceptable postoperative alignment ranging from ≤5 to ≤10 prism diopters (PD), depending on the original study definition. The variability in success definitions was considered during data interpretation and sensitivity analyses.

Secondary objectives were to compare the two surgical approaches regarding postoperative exodrift, operative time, surgical dose-response relationship, and stereopsis. Through these analyses, the study seeks to clarify differences in postoperative alignment and functional outcomes between plication and resection, thereby supporting evidence-based surgical decision-making in exotropia.

Methods

This study adhered to the Preferred Reporting Items for Systematic Reviews and Meta-Analyses (PRISMA) 2020 statement and the Cochrane Handbook for Systematic Reviews of Interventions guidelines [11-13]. The review protocol was prospectively registered in the International Prospective Register of Systematic Reviews (PROSPERO) (#CRD420261326761). The review commenced on September 19, 2025. The initial literature search was completed on November 10, 2025, followed by an updated search on February 27, 2026, before completion of the final analyses.

Eligibility criteria

Population

Studies including pediatric or adult patients with exotropia requiring first-time horizontal rectus surgery involving medial rectus strengthening were eligible. Participants were required to have a measurable exodeviation at distance fixation (typically ≥10-15 prism diopters), encompassing any subtype of exotropia.

Studies enrolling mixed populations of esotropia and exotropia were eligible only if data for patients with exotropia were reported separately. Exclusion criteria included prior strabismus surgery, paralytic or restrictive strabismus, significant vertical deviations, oblique muscle surgery, neurological or craniofacial disorders, and severe amblyopia.

Interventions

The intervention of interest was medial rectus muscle plication, performed either alone or in combination with lateral rectus recession. Plication involves folding the muscle without disinsertion to achieve a muscle-strengthening effect comparable to resection while preserving anterior ciliary circulation.

Comparators

The comparator was conventional medial rectus resection, performed unilaterally or bilaterally, with or without concomitant lateral rectus recession. Studies evaluating modified techniques, including muscle advancement, tucking without formal resection, adjustable-suture resection, or other non-standard procedures, were excluded.

Study design

Only randomized controlled trials (RCTs) were eligible. Randomization was verified through full-text assessment of each study. Two reviewers independently assessed sequence generation, allocation concealment, and other methodological domains using the Cochrane Risk-of-Bias tool. Studies with insufficient reporting of randomization procedures were retained but classified as having an unclear risk of bias where appropriate. We excluded non-randomized studies (including observational studies, retrospective analyses, case series, and quasi-experimental studies), studies without clearly defined outcomes, unpublished studies, conference abstracts, and non-English publications.

Information sources, search strategy, and screening

We conducted a comprehensive literature search of MEDLINE (via PubMed), Web of Science, Scopus, the Cochrane Central Register of Controlled Trials (CENTRAL), and Google Scholar. We considered eligible studies published from database inception to November 10, 2025. Before completion of the final analyses, an updated search was conducted on February 27, 2026, to identify any newly published eligible studies. The search was restricted to articles published in English, with no geographic limitations.

The search strategy combined the following terms related to strabismus, medial rectus strengthening procedures, and surgical outcomes: ((esotropia OR esotrop OR exotropia OR exotrop* OR strabismus OR strabism* OR convergent strabismus OR convergen* OR divergent strabismus OR divergen* OR internal strabismus OR external strabismus OR exodeviation OR divergent squint) AND (resection OR plication OR tucking OR symmetry surgery OR asymmetry surgery OR selective surgery OR bilateral plication OR bilateral resection OR unilateral plication OR unilateral resection OR recess-resect OR recess-plication) AND (treatment outcome OR surgical outcome OR surgical success OR recurrence OR success)).*

Esotropia-related search terms were intentionally included to maximize search sensitivity because some publications include mixed populations of esotropia and exotropia. During study selection, only studies reporting separate data for patients with exotropia were considered eligible.

The search strategy was adapted for each database. Detailed search strategies for all databases are provided in appendix. All retrieved references were imported into Rayyan version 5.4 (Cambridge, MA: Rayyan Systems Inc.) for duplicate removal. The screening process was conducted in two stages. First, two independent reviewers screened titles and abstracts, with disagreements resolved by a third reviewer. Second, two independent reviewers assessed the full texts of potentially eligible studies, and a senior author verified all inclusion and exclusion decisions. Disagreements were resolved through discussion until consensus was reached. Reference lists of all included studies and relevant review articles were also manually searched to identify additional eligible studies.

Data extraction

Two reviewers independently extracted data using a predefined standardized data extraction form. Extracted information included study characteristics, participant demographics, surgical procedures, comparator interventions, duration of follow-up, and outcome measures, including surgical success, postoperative deviation change, postoperative exodrift, operative time, surgical dose-response relationship, and stereopsis. A senior reviewer independently verified all extracted data and resolved discrepancies through discussion. When necessary, corresponding authors were contacted to clarify unclear or missing information. Missing data were not imputed.

Risk-of-bias assessment in the individual studies and between the published studies

Risk of bias was assessed independently by two reviewers using the Cochrane Risk-of-Bias (RoB 1) tool, implemented in RevMan Web 2026 (London, UK: Cochrane Collaboration) [13]. We evaluated the following domains: sequence generation, allocation concealment, blinding of participants and personnel, blinding of outcome assessment, incomplete outcome data, selective outcome reporting, and other potential sources of bias.

Particular attention was paid to the adequacy of random sequence generation and allocation concealment to confirm the randomized design of each included trial. Studies with insufficient methodological reporting were classified as having an unclear risk of bias in the corresponding domains rather than being excluded. Disagreements between reviewers were resolved through discussion and re-evaluation of the original reports.

The risk-of-bias assessment was incorporated into the interpretation of pooled results and sensitivity analyses. Studies judged to have a high risk of bias in one or more domains were retained because of the limited number of eligible randomized controlled trials; however, sensitivity analyses were performed to evaluate their influence on the pooled estimates. Publication bias was not assessed using funnel plots or Egger's regression because fewer than 10 studies were available for each meta-analysis.

Certainty of evidence (GRADE assessment)

We assessed the certainty of evidence using the Grading of Recommendations Assessment, Development and Evaluation (GRADE) approach, initially classifying randomized controlled trials as high certainty. We then downgraded these based on factors such as risk of bias, inconsistency, indirectness, imprecision, and publication bias. Downgrading occurred when studies showed methodological limitations, heterogeneity, indirect applicability, wide confidence intervals, or potential selective reporting.

Data analysis

Statistical analyses were performed using Review Manager (RevMan Web 2026; London, UK: Cochrane Collaboration) [13]. For dichotomous outcomes (surgical success and stereopsis), the inverse-variance method was used to calculate risk ratios (RRs) with 95% confidence intervals (CIs). The inverse-variance approach was selected because the analyses were performed using a random-effects model, for which both inverse-variance and Mantel-Haenszel methods are appropriate according to the Cochrane Handbook. To confirm the robustness of the findings, we repeated all dichotomous analyses using the Mantel-Haenszel method, yielding identical overall conclusions (appendix).

For continuous outcomes (postoperative deviation change, postoperative exodrift, operative time, and surgical dose-response relationship), mean differences (MDs) with 95% confidence intervals were calculated. All analyses were performed using the final reported follow-up for each study, with statistical significance defined as p<0.05.

Statistical heterogeneity was evaluated using the chi-square (χ²) test and the I² statistic, with values of <25%, 25-50%, and >50% representing low, moderate, and substantial heterogeneity, respectively. Random-effects meta-analysis using the restricted maximum likelihood (REML) estimator was prespecified because clinical and methodological heterogeneity among studies was anticipated.

Potential sources of heterogeneity were explored through predefined subgroup analyses based on exotropia subtype and preoperative deviation magnitude, together with leave-one-out sensitivity analyses. Studies producing substantial changes in heterogeneity were not considered statistical outliers but were examined for potential clinical and methodological differences that could explain divergent findings. The Hartung-Knapp-Sidik-Jonkman (HKSJ) adjustment was applied to obtain more conservative confidence intervals. For studies with zero events in one treatment arm, a continuity correction was applied. Only relevant treatment arms from multiarm studies were included. All analytical decisions were predefined in the review protocol. All data analyzed during this study are presented in the article and in appendix.

Results

Literature Search Results

The literature search identified 7,425 records from five electronic databases (PubMed: n=1,469; Web of Science: n=2,783; Scopus: n=1,975; Cochrane CENTRAL: n=3; and Google Scholar: n=1,195). After removal of 2,694 duplicates, 4,731 unique records remained for title and abstract screening. A total of 4,721 records were excluded because they were irrelevant, non-comparative studies, non-English publications, involved ineligible populations, or did not meet the predefined eligibility criteria. A total of 10 full-text articles were assessed for eligibility, of which five randomized controlled trials met the inclusion criteria.

Following prospective registration of the review protocol in PROSPERO (#CRD420261326761), an updated literature search was performed on February 27, 2026, before completion of the final analyses. This search identified one additional eligible randomized controlled trial (Atalay and Koç), which fulfilled the predefined eligibility criteria and was incorporated into the quantitative synthesis [14]. No additional eligible studies were identified through manual screening of reference lists. Ultimately, six randomized controlled trials involving 352 participants were included in the systematic review and meta-analysis. The study selection process is presented in Figure 1 [14].

**Figure 1:**
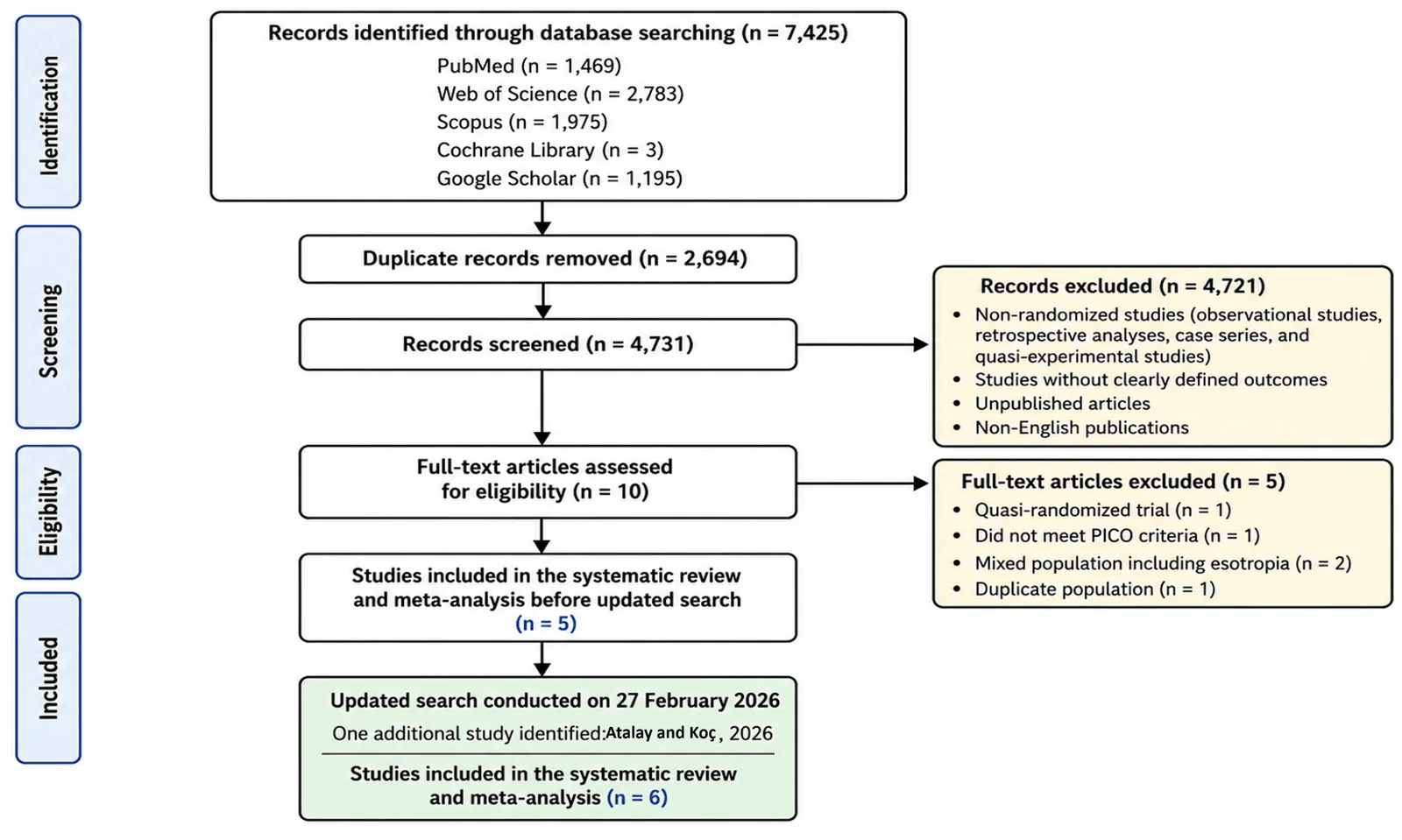
PRISMA flow diagram included searches of databases. PRISMA: Preferred Reporting Items for Systematic Reviews and Meta-Analyses; PICO: Population, Intervention, Comparison, and Outcome

Characteristics of the Included Studies

The meta-analysis included six randomized controlled trials comprising 352 participants, including 175 patients who underwent medial rectus plication and 177 who underwent medial rectus resection. Baseline demographic and clinical characteristics were generally comparable between treatment groups.

The included studies evaluated different clinical subtypes of exotropia, including basic intermittent exotropia, convergence insufficiency-type intermittent exotropia, sensory exotropia, concomitant exotropia, and large-angle exotropia, with varying preoperative deviation angles and follow-up durations (Tables 1, 2). Definitions of surgical success varied among studies, with acceptable postoperative alignment defined as residual deviations ranging from ≤5 to ≤10 prism diopters. Reporting of sensory outcomes, including stereopsis, was inconsistent across studies, limiting quantitative synthesis of functional outcomes.

**Table 1:** Summary of the included studies. PD: prism diopters; IXT: intermittent exotropia; MR: medial rectus; LR: lateral rectus; XT: exotropia; BLR: bilateral lateral rectus

Studies	Design	Population	Total, n	Key intervention	Follow-up (months)	Main findings
Wang et al. (2019) [15]	Prospective RCT	CI-type IXT (near greater than distance by 10 PD or more), exodeviation 15 PD or more at distance	55	Bilateral MR plication (P) versus bilateral MR resection (R)	6	Less immediate overcorrection with plication; similar success at 6 months. Shorter operative time, simpler, safer, and less traumatic.
Anand et al. (2020) [16]	Prospective RCT	Basic IXT (35-55 PD)	60	8 mm LR recession+MR plication versus resection	12	Comparable 12-month success, exodrift, and dose effect. With plication having a certain added advantage.
Daga et al. (2022) [17]	Prospective RCT	Intermittent, constant, and sensory XT	80	LR recession+reinforced MR plication versus resection	6	Similar deviation correction and exodrift.
Hegazy and Mansour (2024) [10]	Prospective RCT	Basic IXT	27	LR recession+MR plication versus resection	3	Comparable success; preoperative control predicted outcome. Plication has additional benefits.
Elsayed et al. (2025) [8]	Prospective RCT	Large-angle XT ≥60 PD	40	BLR recession+unilateral MR plication versus resection	6	Similar success; shorter operative time with plication.
Atalay and Koç (2026) [14]	Prospective RCT	Basic intermittent XT ≥20 PD; age ≥9 years. IXT control scores 4 or higher for either distant or near fixation	90	MR plication+LR recession versus MR resection+LR recession	12	1-year success: 55% (P) vs 80% (R); significantly greater exodrift and undercorrection in plication group.

**Table 2:** Baseline characteristics of included studies. PD: prism diopters; IXT: intermittent exotropia; XT: exotropia; ET: esotropia; P: plication; R: resection

Studies	Country	Sample (P/R)	Mean age (years)	Sex (M/F)	Mean preoperative deviation (PD)	Inclusion criteria	Outcomes
Wang et al. (2019) [15]	China	27/28	P: 10.4±6.6, R: 9.1±5.6	31/24 (P: 16/11, R: 15/13)	Dist: P: 33.7±12.6, R: 36.1±11.0, Near P: -49.4±13.5, R: -52.4±11.9	CI-type IXT; near-distance disparity ≥10 PD; distance deviation ≥15 PD	Success within ±10 PD; near-distance difference; stereoacuity
Anand et al. (2020) [16]	India	30/30	P: 20.7±9.2, R: 19.1±8.3	31/29 (P: 15/15, R: 16/14)	P: 44.7±4.5, R: 43.2±4.8	Age >6 years; basic-type intermittent XT; deviation 35-55 PD	Success ±10 PD at 12 months and exodrift
Daga et al. (2022) [17]	India	41/39	P: 23.3±10, R: 23.5±11.9	44/36 (P: 23/18, R: 21/18)	~50 PD Distance P: 50.12±9.8, R: 50.2±11.9, Near P: 50.0±10.2, R: 51.3±10.0	Comitant XT ≥15 PD	Postoperative deviation; exodrift
Hegazy and Mansour (2024) [10]	Egypt	13/14	P: 26.5±9.5, R: 26.0±12.3	7/20 (P: 2/11, R: 5/9)	~55 PD (P: 53.9±11.6, R: 56.1±9.6)	Age 10-45 years; basic IXT (near-distance difference <10 PD)	Success: <10 PD XT/<5 PD ET
Elsayed et al. (2025) [8]	Egypt	20/20	P: 23.9±8.1, R: 24.0±8.7	20/20 (P: 11/9, R: 9/11)	~72 PD (P: 71.8±7.9, R: 72.0±8.8)	Age 15-45 years; large-angle concomitant (intermittent or constant) XT ≥60 PD; no prior surgery	Success ≤10 PD XT/≤5 PD ET
Atalay and Koç (2026) [14]	Turkey	44/46	P: 26.7±15.4, R: 26.3±14.5	P: 24/20, R: 27/19	P: 38.5±8.7, R: 40.8±10.5	Age ≥9 years; IXT ≥20 PD; control score ≥4, not having any other eye comorbidity except amblyopia and pseudophakia	Success ±10 PD at 12 months; exodrift; recurrence

Assessment and management of clinical heterogeneity

Clinical heterogeneity was explored using predefined subgroup analyses according to exotropia subtype and preoperative deviation angle, together with leave-one-out sensitivity analyses. Statistical heterogeneity was generally low to moderate for most pooled outcomes but increased in selected subgroup analyses owing to differences in patient characteristics and study populations. The findings of subgroup analyses should therefore be interpreted cautiously because of the limited number of included studies.

Risk of bias

Overall, the included studies demonstrated acceptable methodological quality. Most studies were judged to be at low risk of bias for random sequence generation, incomplete outcome data, and selective reporting. Allocation concealment and blinding of participants, personnel, and outcome assessment were frequently reported as unclear because of insufficient methodological detail. One study was judged to have a high risk of detection bias owing to lack of blinding of outcome assessment. The overall risk-of-bias assessment is presented in Figure 2.

**Figure 2:**
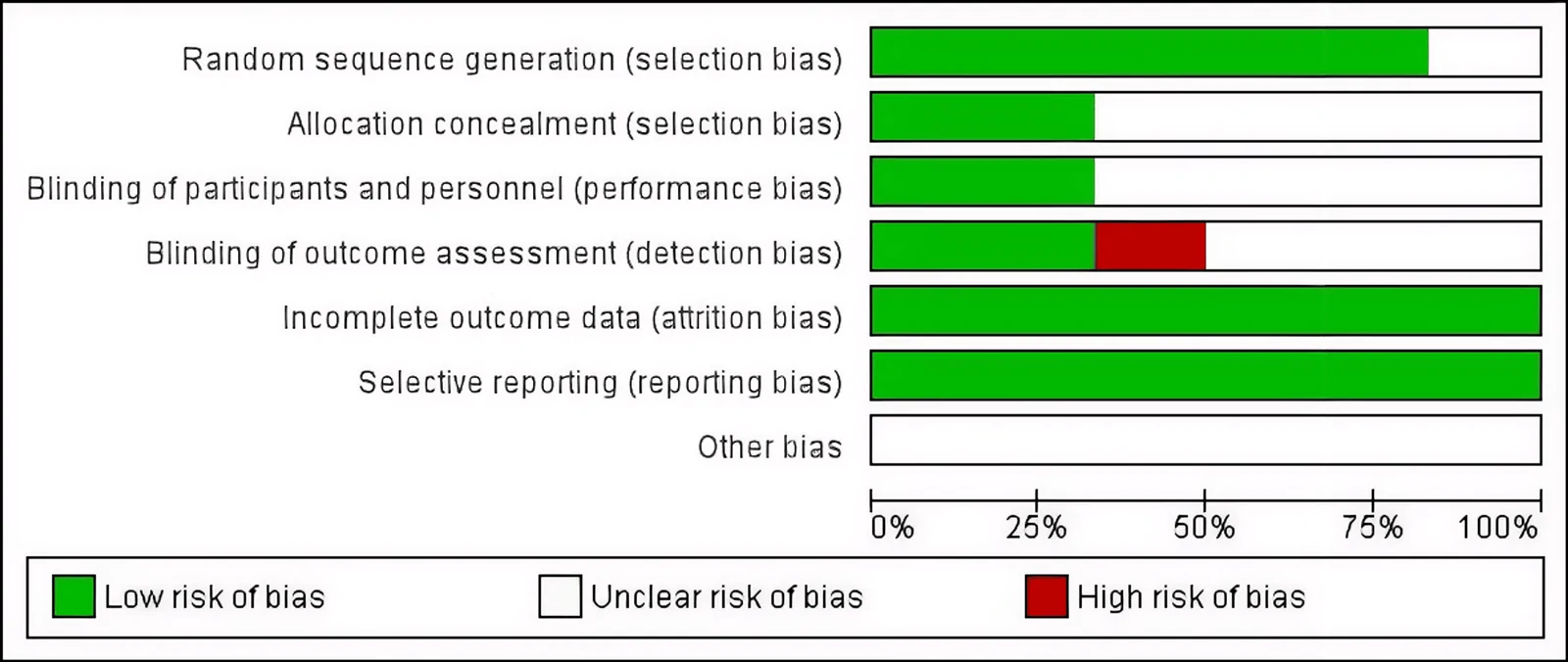
Risk-of-bias assessment of the studies.

Surgical Success Rate

Five studies involving 268 participants (132 undergoing medial rectus plication and 136 undergoing medial rectus resection) reported surgical success. The pooled analysis demonstrated no statistically significant difference between the two procedures (RR=0.86, 95% CI: 0.66-1.14; p=0.22), with moderate heterogeneity that was not statistically significant (I²=40%, p=0.17) (Figure 3) [8,10,14-16]. Sensitivity analysis excluding the study by Atalay and Koç shifted the pooled estimate toward equivalence (RR=0.96, 95% CI: 0.82-1.11; p=0.41) and eliminated heterogeneity (I²=0%) (Figure 4) [8,10,14-16]. Subgroup analysis according to strabismus type showed no statistically significant differences within any subgroup or in the overall test for subgroup differences (p=0.62). Likewise, subgroup analysis based on preoperative deviation magnitude revealed no statistically significant differences either within individual subgroups or between subgroups (Figures 5, 6) [8,10,14-16].

**Figure 3:**
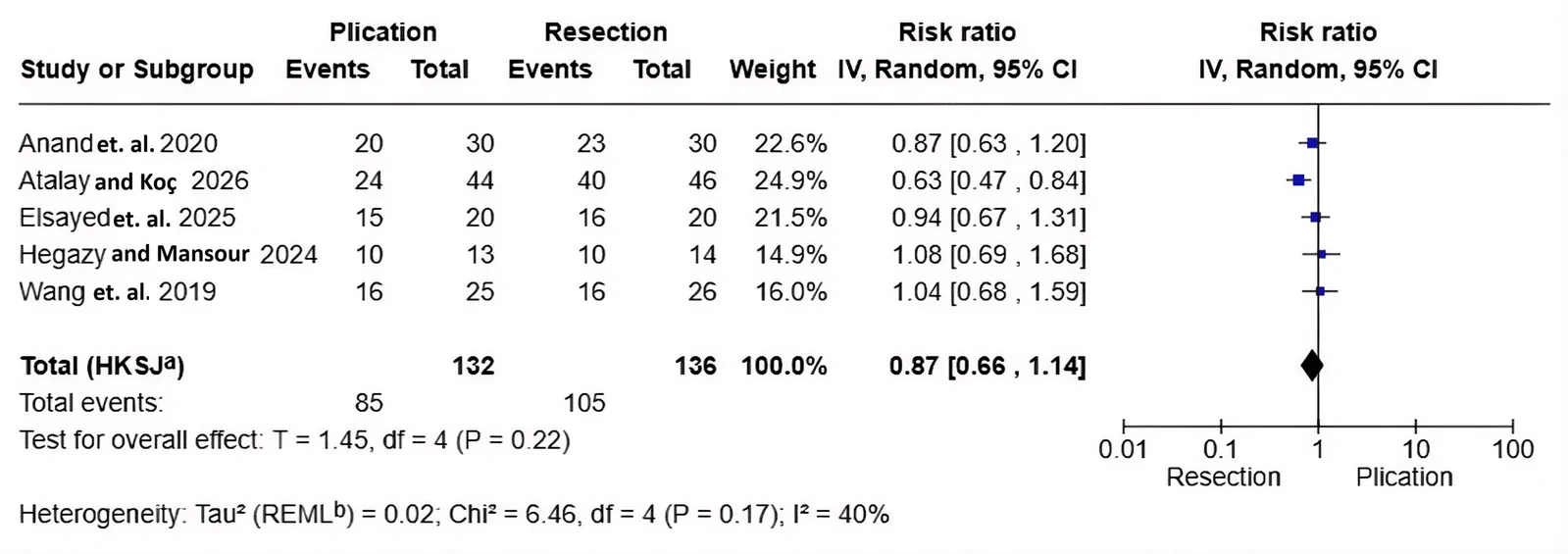
Forest plot of RCTs comparing MR plication with resection for success rate. ^a^Confidence interval calculated using the Hartung-Knapp-Sidik-Jonkman (HKSJ) method. ^b^Tau² calculated using the restricted maximum-likelihood (REML) method. MR: medial rectus; REML: restricted maximum likelihood; HKSJ: Hartung-Knapp-Sidik-Jonkman; IV: inverse variance

**Figure 4:**
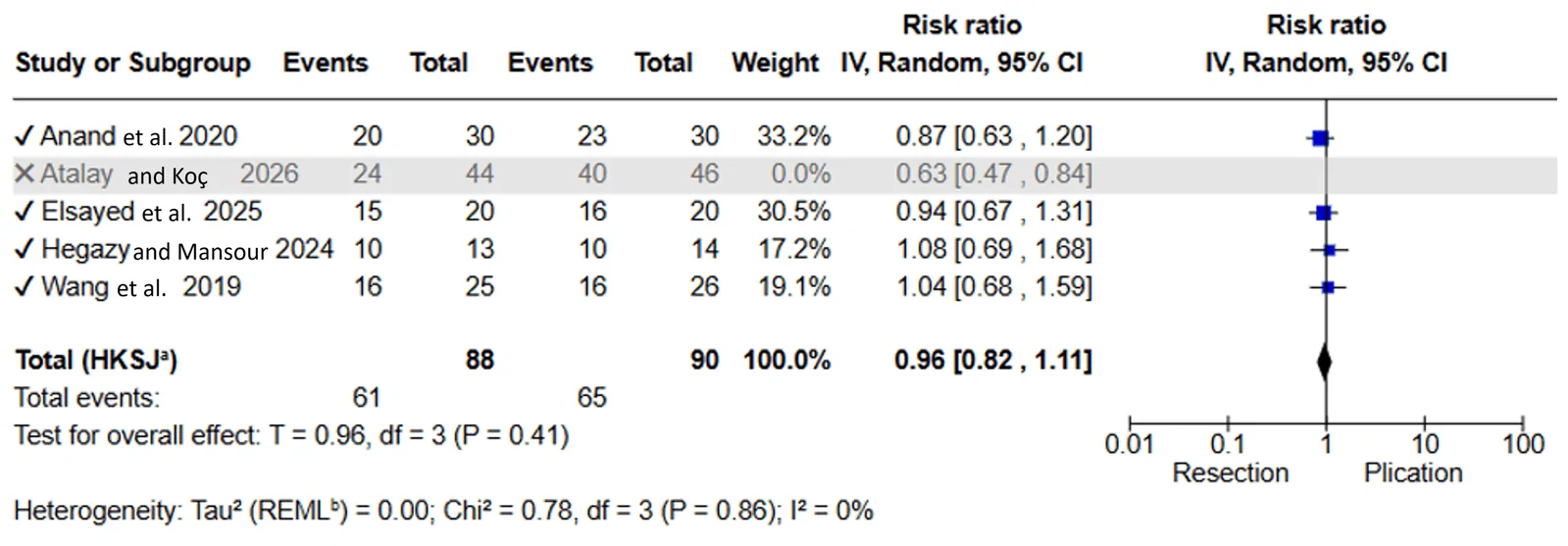
Sensitivity analysis of surgical success comparing medial rectus plication with resection, excluding Atalay and Koç, shows no significant difference. ^a^Confidence interval calculated using the Hartung-Knapp-Sidik-Jonkman (HKSJ) method. ^b^Tau² calculated using the restricted maximum-likelihood (REML) method. REML: restricted maximum likelihood; HKSJ: Hartung-Knapp-Sidik-Jonkman; IV: inverse variance

**Figure 5:**
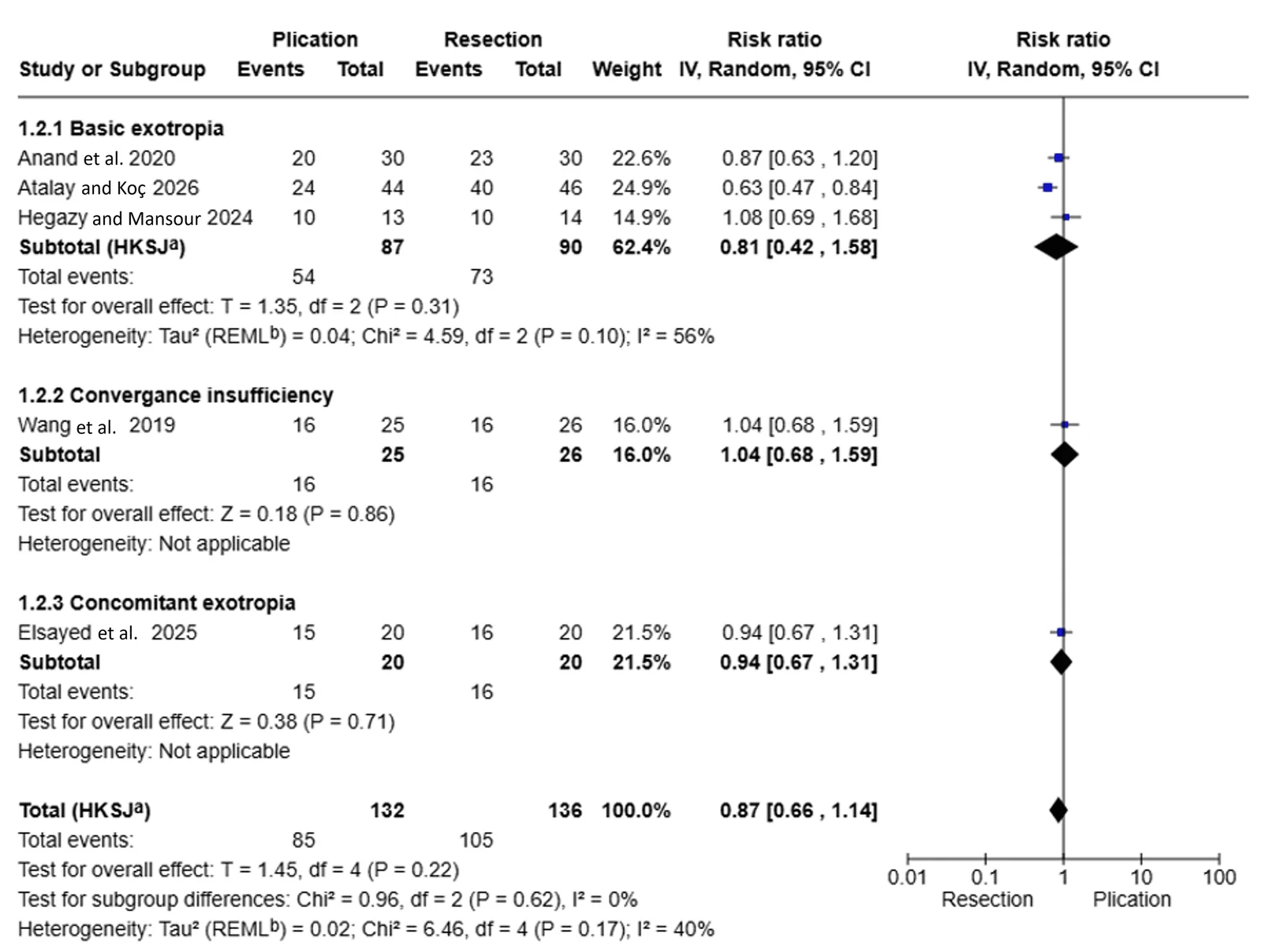
Subgroup analysis by exotropia type showed no statistically significant differences between medial rectus plication and medial rectus resection across any subtype, with no significant subgroup effect. ^a^Confidence interval calculated using the Hartung-Knapp-Sidik-Jonkman (HKSJ) method. ^b^Tau² calculated using the restricted maximum-likelihood (REML) method. REML: restricted maximum likelihood; HKSJ: Hartung-Knapp-Sidik-Jonkman; IV: inverse variance

**Figure 6:**
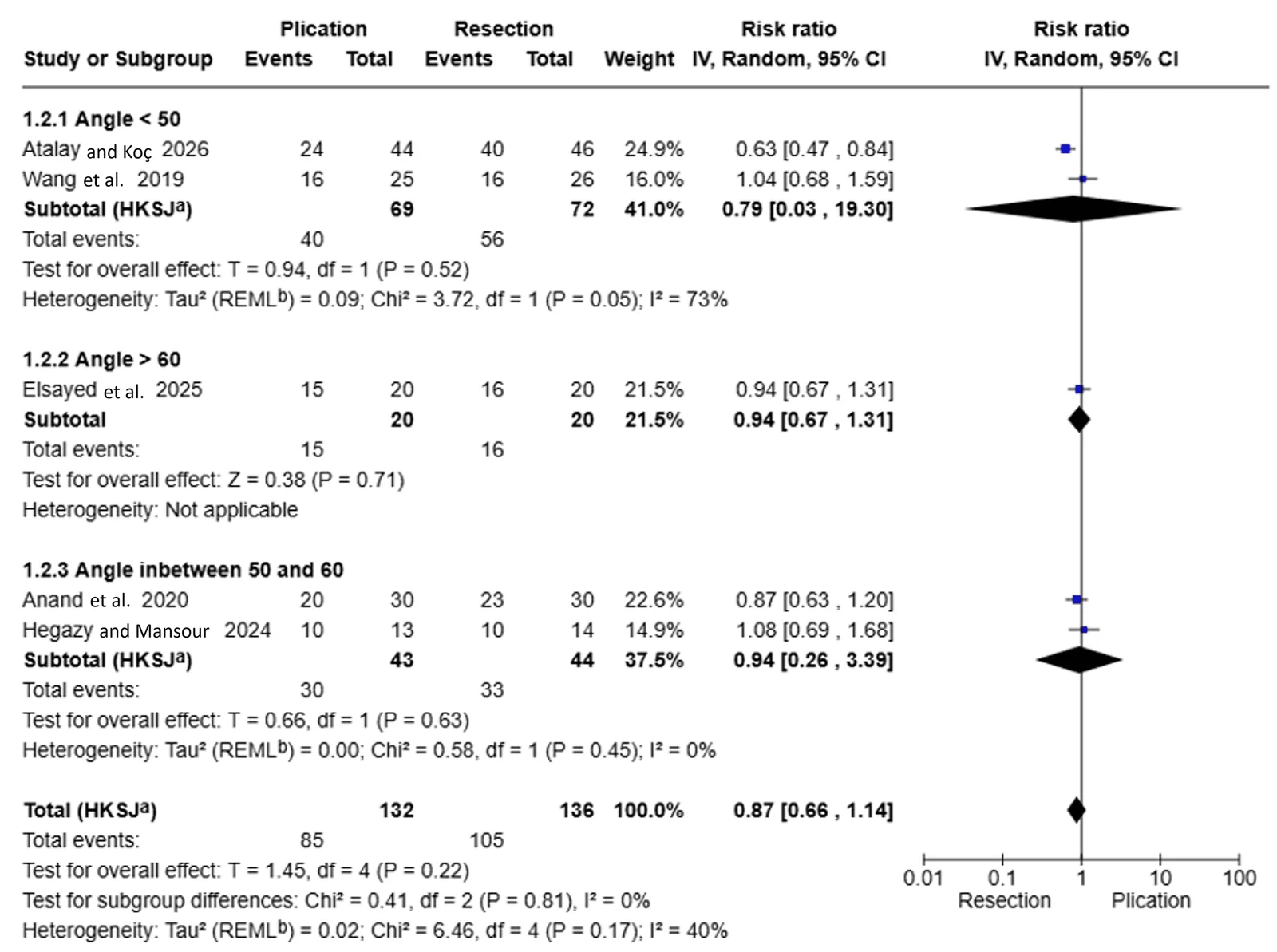
Subgroup analysis by preoperative deviation magnitude showed no statistically significant differences between medial rectus plication and medial rectus resection across any deviation category, with no significant subgroup effect. ^a^Confidence interval calculated using the Hartung-Knapp-Sidik-Jonkman (HKSJ) method. ^b^Tau² calculated using the restricted maximum-likelihood (REML) method. REML: restricted maximum likelihood; HKSJ: Hartung-Knapp-Sidik-Jonkman; IV: inverse variance

Postoperative Deviation Change

Six studies (173 MR plication, 175 MR resection) reported postoperative deviation change. The pooled analysis showed no statistically significant difference between the two techniques (MD=0.84 PD, 95% CI: -2.51 to 4.20; p=0.55), with moderate heterogeneity that was not statistically significant (I²=41%; p=0.18) (Figure 7) [8,10,14-17]. Sensitivity analysis excluding Atalay and Koç eliminated heterogeneity (I²=0%) without altering the overall non-significant pooled estimate (Figure 8) [8,10,14-17].

**Figure 7:**
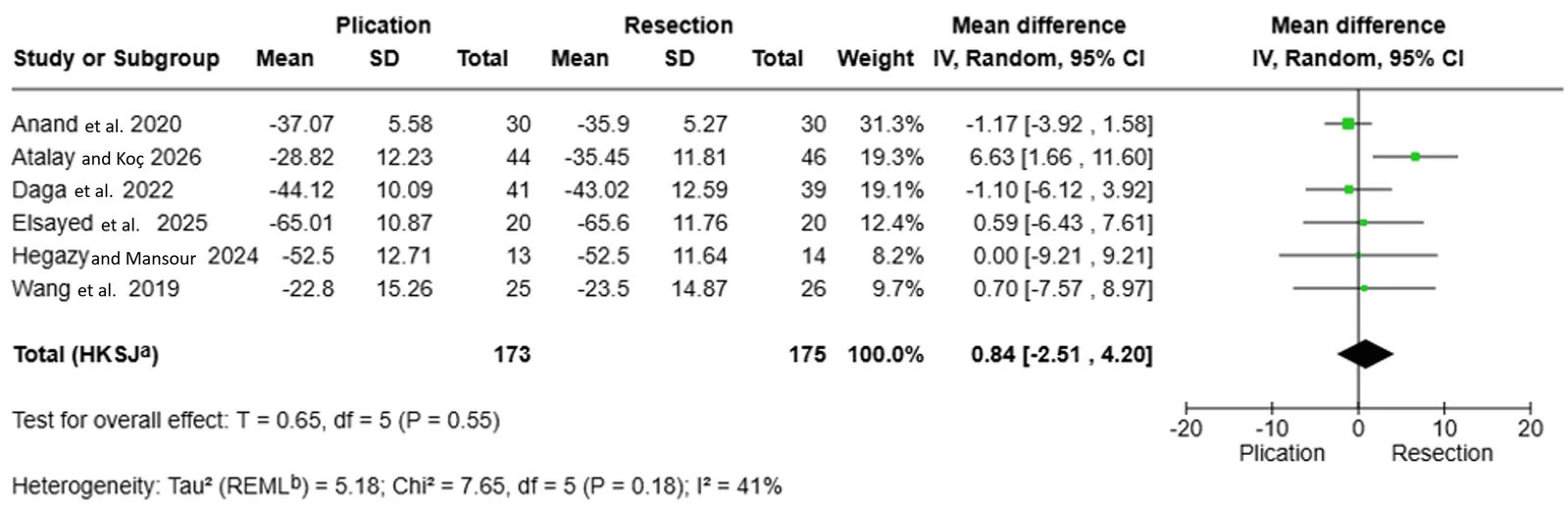
Forest plot comparing postoperative angle deviation change between MR plication and resection. The pooled analysis shows no significant difference. ^a^Confidence interval calculated using the Hartung-Knapp-Sidik-Jonkman (HKSJ) method. ^b^Tau² calculated using the restricted maximum-likelihood (REML) method. MR: medial rectus; REML: restricted maximum likelihood; HKSJ: Hartung-Knapp-Sidik-Jonkman; IV: inverse variance

**Figure 8:**
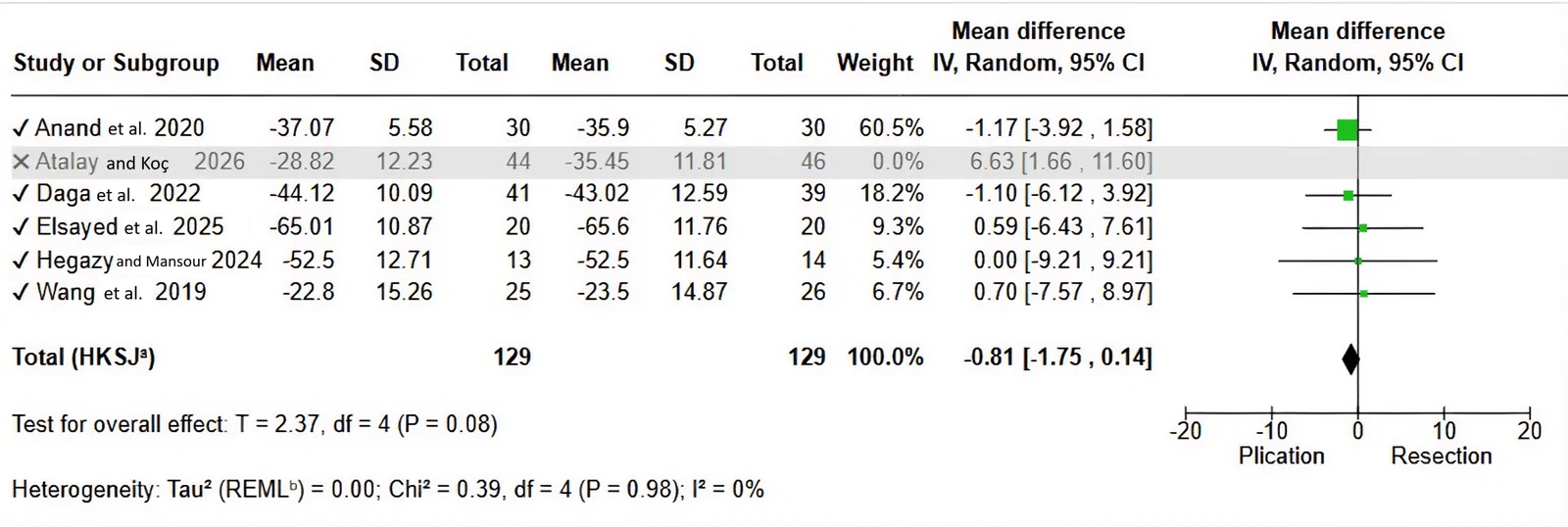
Sensitivity analysis of postoperative deviation shows no significant difference between plication and resection. ^a^Confidence interval calculated using the Hartung-Knapp-Sidik-Jonkman (HKSJ) method. ^b^Tau² calculated using the restricted maximum-likelihood (REML) method. REML: restricted maximum likelihood; HKSJ: Hartung-Knapp-Sidik-Jonkman; IV: inverse variance

Subgroup analyses according to strabismus type and preoperative deviation magnitude generally showed no statistically significant differences. Although the 50-59 PD subgroup demonstrated a significantly smaller postoperative deviation change following MR plication compared with MR resection (MD= -1.08 PD, 95% CI: -1.94 to -0.22; p=0.03), this finding was not observed in the remaining subgroups. Moreover, the test for subgroup differences according to preoperative deviation magnitude was not statistically significant (p=0.12). Therefore, these subgroup findings should be considered exploratory and interpreted with caution because of the limited number of included studies (Figures 9, 10) [8,10,14-17].

**Figure 9:**
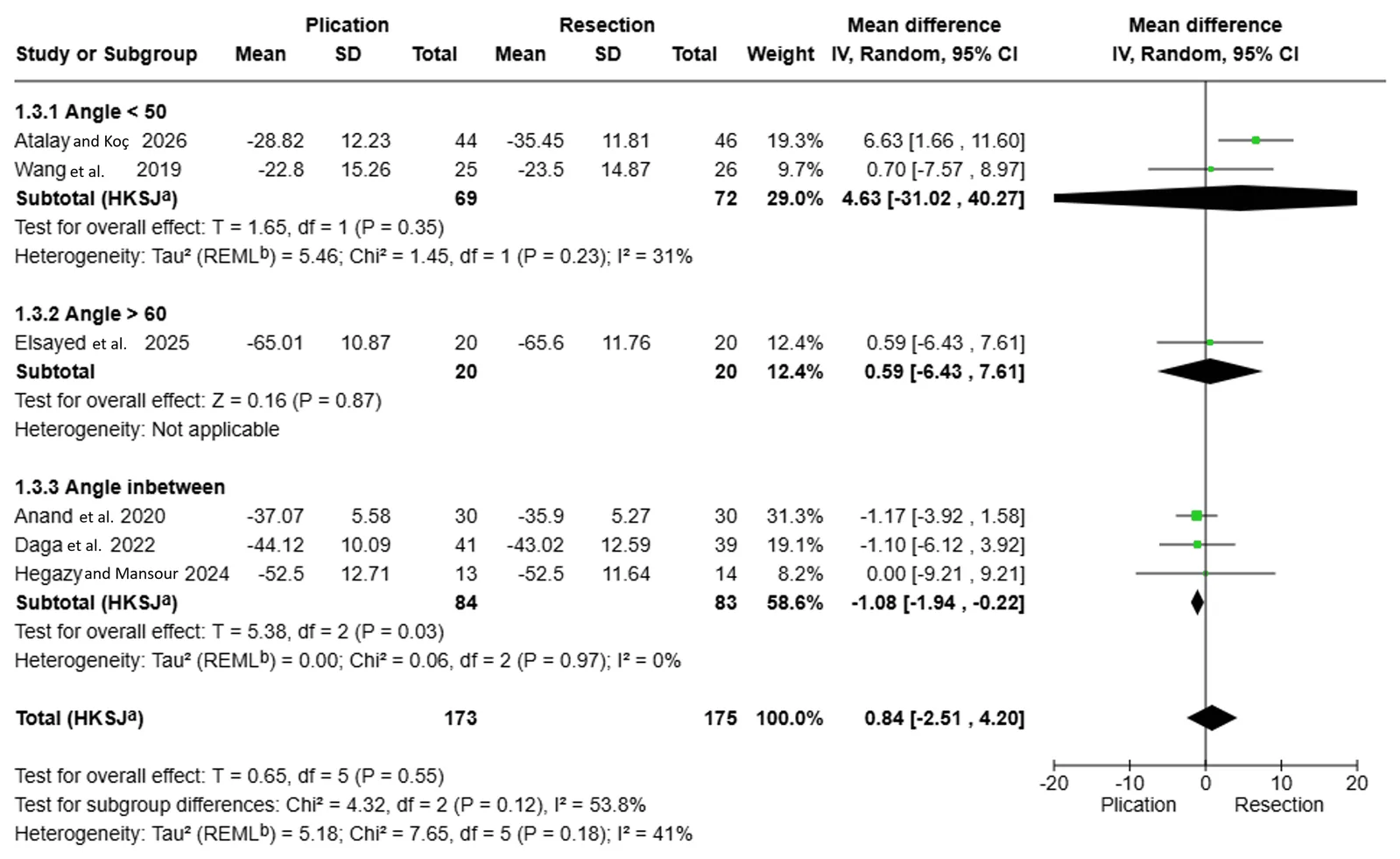
Subgroup analysis of postoperative deviation by preoperative angle shows no overall difference between plication and resection, with a significant effect favoring resection in the <50 PD subgroup only. ^a^Confidence interval calculated using the Hartung-Knapp-Sidik-Jonkman (HKSJ) method. ^b^Tau² calculated using the restricted maximum-likelihood (REML) method. PD: prism diopters; REML: restricted maximum likelihood; HKSJ: Hartung-Knapp-Sidik-Jonkman; IV: inverse variance

**Figure 10:**
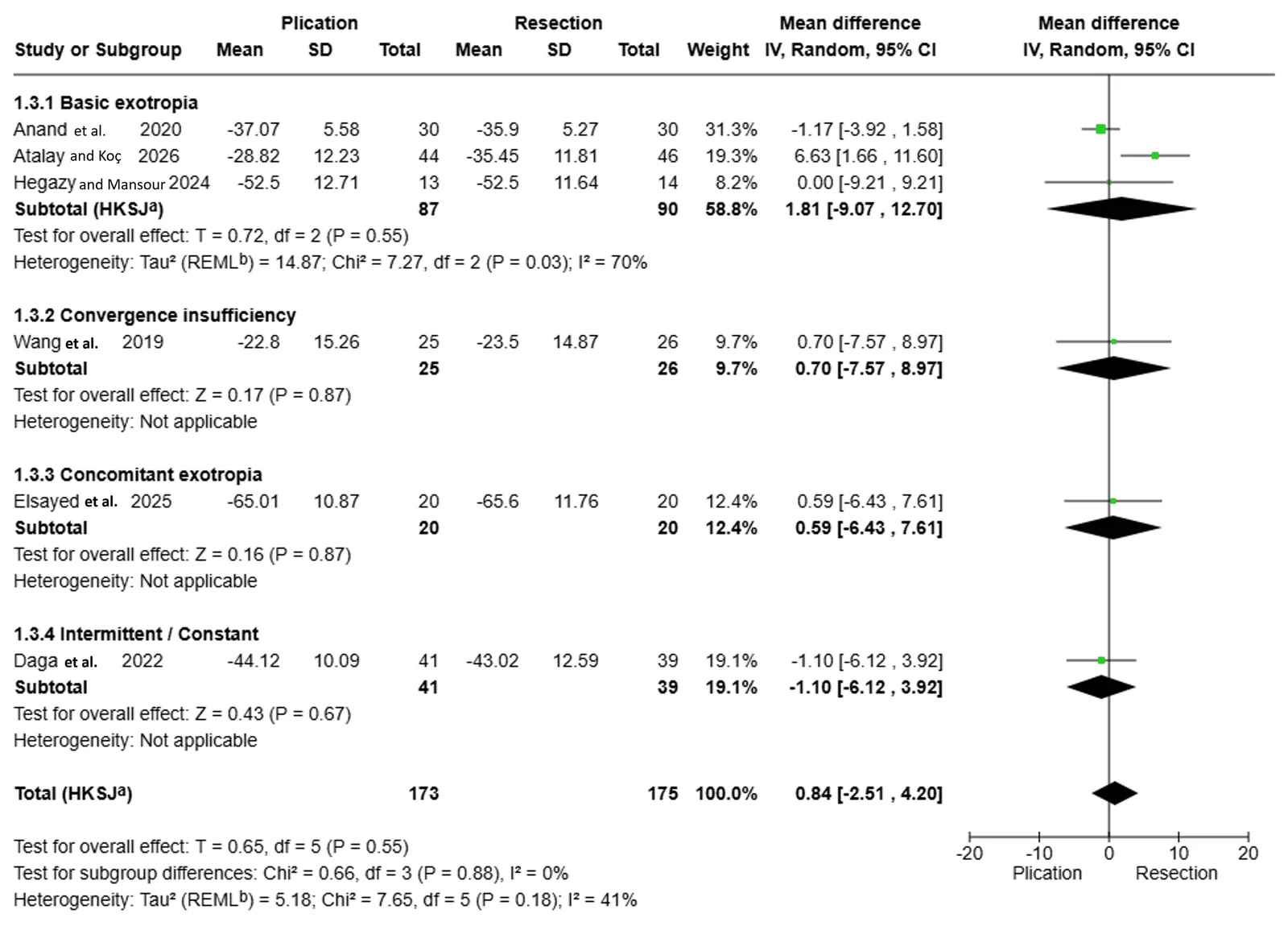
Subgroup analysis of postoperative deviation by exotropia type shows no significant difference between plication and resection across all subtypes or overall. ^a^Confidence interval calculated using the Hartung-Knapp-Sidik-Jonkman (HKSJ) method. ^b^Tau² calculated using the restricted maximum-likelihood (REML) method. REML: restricted maximum likelihood; HKSJ: Hartung-Knapp-Sidik-Jonkman; IV: inverse variance

Postoperative Exodrift

Four studies (281 patients) reported postoperative exodrift. The pooled analysis showed no statistically significant difference between MR plication and MR resection (MD=1.40 PD, 95% CI: -3.03 to 5.82; p=0.38), despite substantial statistically significant heterogeneity (I²=80%; p=0.004) (Figure 11) [14-17]. Sensitivity analysis indicated that Atalay and Koç were the primary contributors to heterogeneity; exclusion of this study eliminated heterogeneity (I²=0%; p=0.17) while maintaining a non-significant pooled estimate (p=0.81), supporting comparable postoperative exodrift between the two procedures (Figure 12) [14-17]. Subgroup analysis according to preoperative deviation magnitude showed no statistically significant differences within any subgroup. Although the test for subgroup differences was statistically significant (p<0.00001), these findings should be interpreted cautiously because of the limited number of included studies and the substantial influence of Atalay and Koç on the pooled estimates [14].

**Figure 11:**
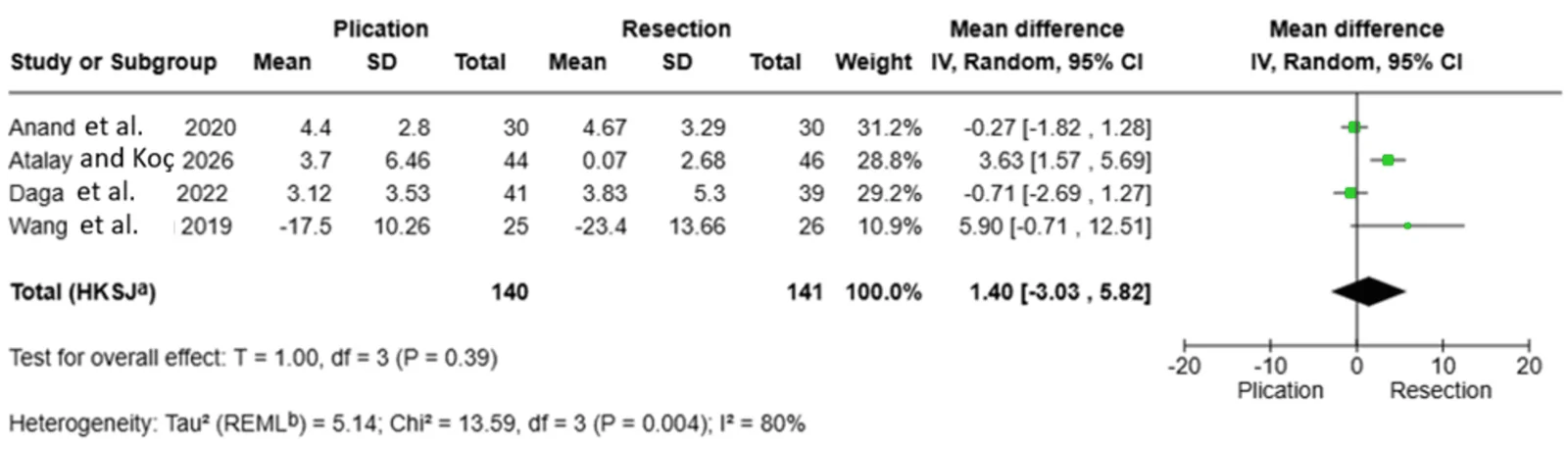
Forest plot comparing exodrift (postoperative drift) between MR plication and resection. The pooled result shows no significant difference. ^a^Confidence interval calculated using the Hartung-Knapp-Sidik-Jonkman (HKSJ) method. ^b^Tau² calculated using the restricted maximum-likelihood (REML) method. MR: medial rectus; REML: restricted maximum likelihood; HKSJ: Hartung-Knapp-Sidik-Jonkman; IV: inverse variance

**Figure 12:**
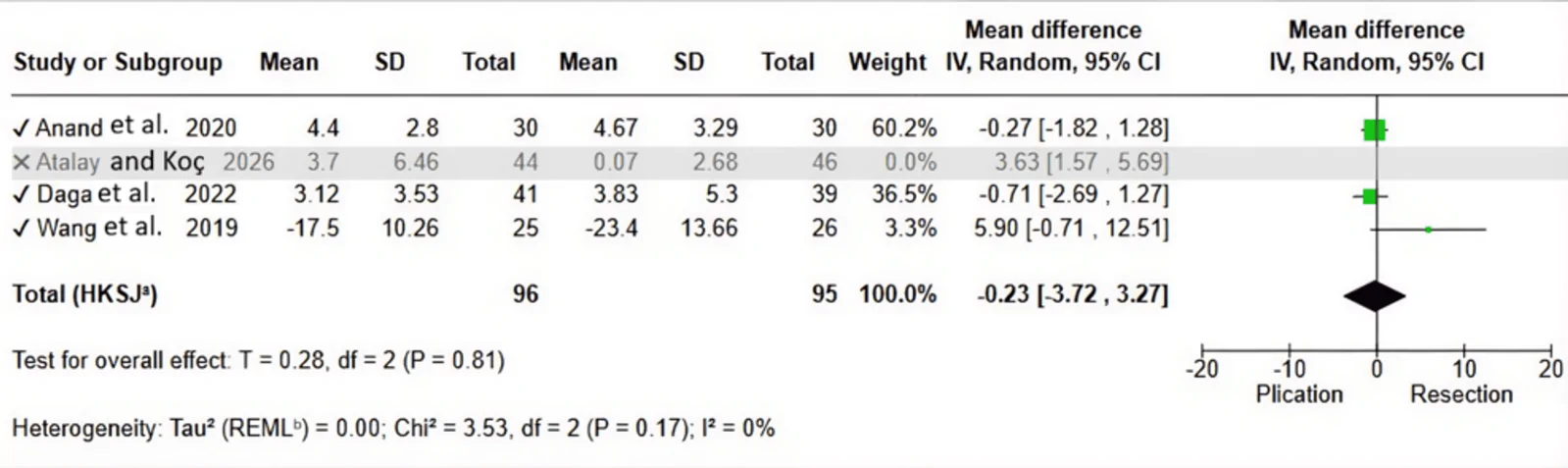
Sensitivity analysis of postoperative exodrift shows no significant difference between plication and resection. ^a^Confidence interval calculated using the Hartung-Knapp-Sidik-Jonkman (HKSJ) method. ^b^Tau² calculated using the restricted maximum-likelihood (REML) method. REML: restricted maximum likelihood; HKSJ: Hartung-Knapp-Sidik-Jonkman; IV: inverse variance

Operative Time

Three studies (155 procedures) reported operative time. The pooled analysis showed no statistically significant difference between MR plication and MR resection (MD= -2.07 min, 95% CI: -7.19 to 3.04; p=0.22), although substantial statistically significant heterogeneity was observed (I²=92%; p=0.002) (Figure 13) [8,15,16]. Sensitivity analysis did not resolve the heterogeneity, as sequential exclusion of individual studies did not materially reduce between-study variability. Individual studies generally suggested shorter operative times with MR plication; however, the persistent heterogeneity likely reflects differences in surgical techniques, surgeon experience, and case complexity.

**Figure 13:**
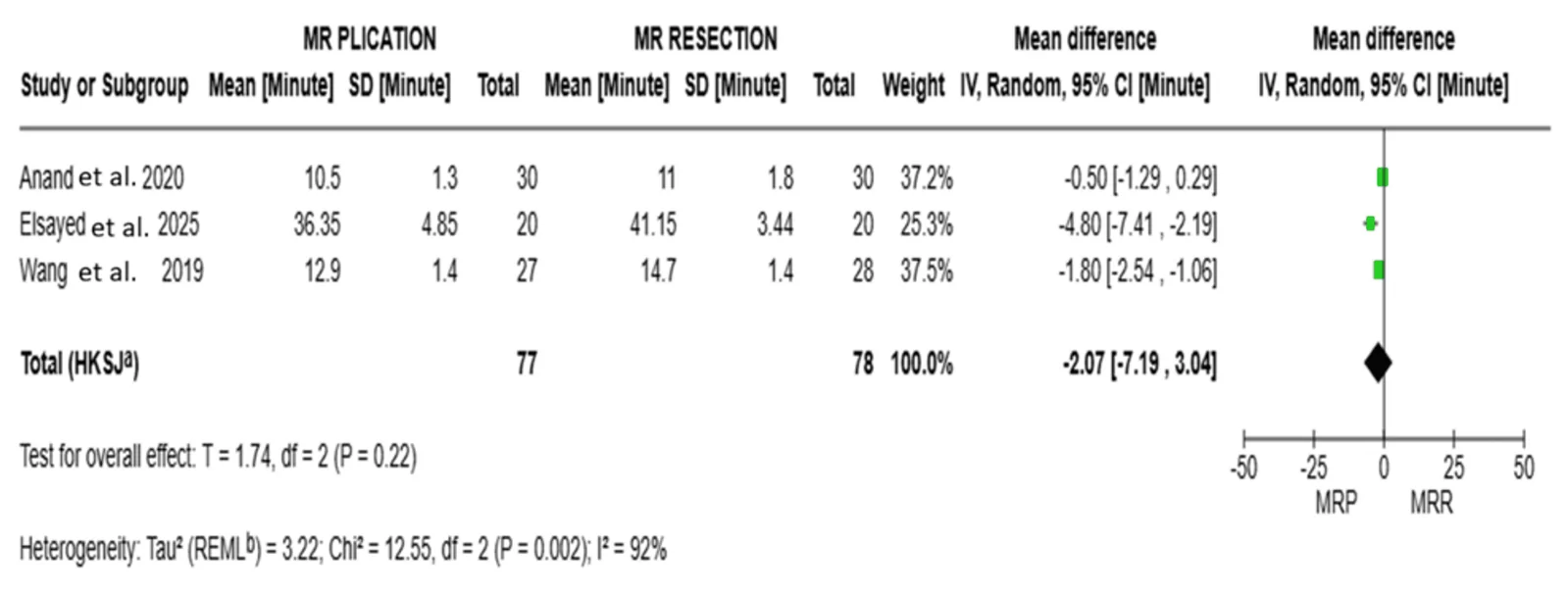
Forest plot comparing operative time between MR plication and resection. The pooled analysis shows no significant difference. ^a^Confidence interval calculated using the Hartung-Knapp-Sidik-Jonkman (HKSJ) method. ^b^Tau² calculated using the restricted maximum-likelihood (REML) method. MR: medial rectus; REML: restricted maximum likelihood; HKSJ: Hartung-Knapp-Sidik-Jonkman; IV: inverse variance; MRP: medial rectus plication; MRR: medial rectus resection

Surgical Dose

Three randomized controlled trials (190 patients) reported postoperative dose-response. The pooled analysis demonstrated a statistically significant difference favoring MR plication (MD= -0.29 PD/mm, 95% CI: -0.48 to -0.09; p=0.02), with no evidence of heterogeneity (I²=0%; p=0.94) (Figure 14) [8,14,16]. However, the magnitude of the observed difference was small and should be interpreted cautiously, as its clinical relevance remains uncertain.

**Figure 14:**
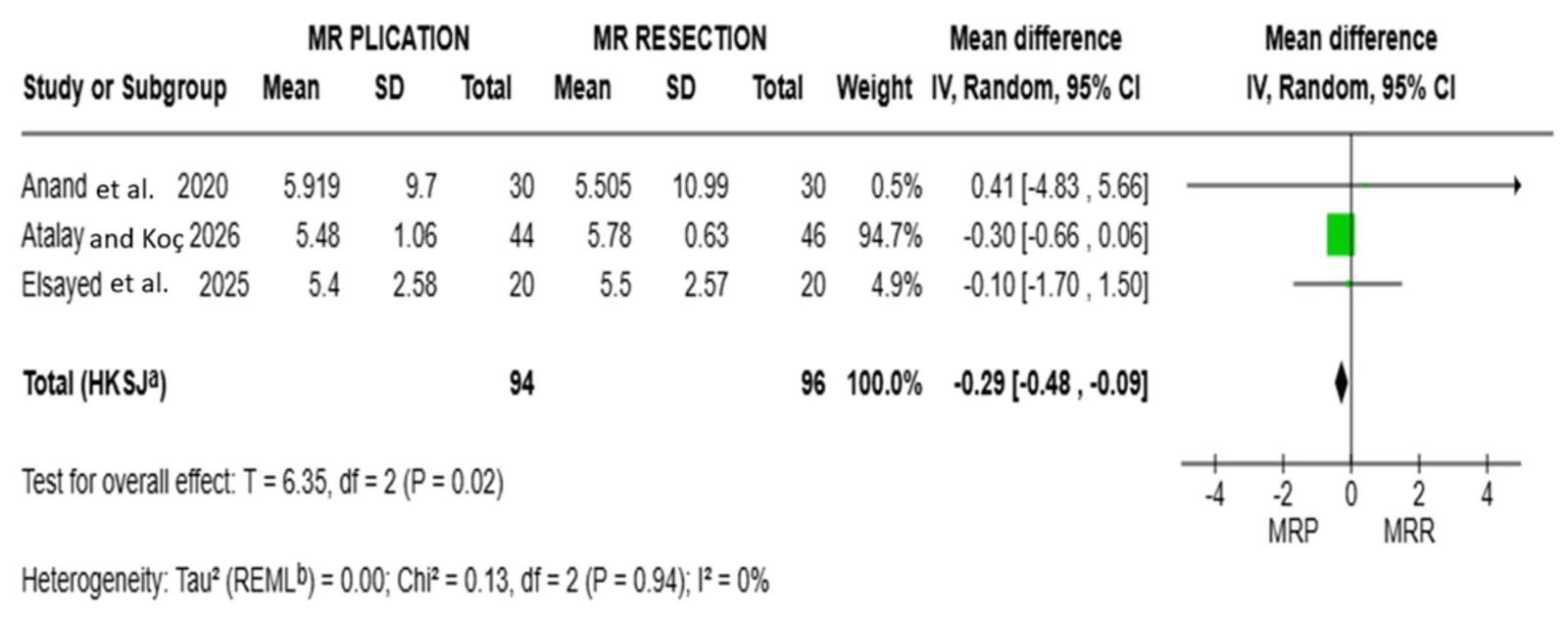
Forest plot comparing dose-response between MR plication and resection. The pooled estimate shows a significant difference favoring plication. ^a^Confidence interval calculated using the Hartung-Knapp-Sidik-Jonkman (HKSJ) method. ^b^Tau² calculated using the restricted maximum-likelihood (REML) method. MR: medial rectus; REML: restricted maximum likelihood; HKSJ: Hartung-Knapp-Sidik-Jonkman; IV: inverse variance; MRP: medial rectus plication; MRR: medial rectus resection

Stereopsis

Three studies (201 participants) reported postoperative stereopsis. The pooled analysis demonstrated a borderline statistically significant difference between MR plication and MR resection (RR=0.88, 95% CI: 0.78-1.00; p=0.05). No heterogeneity was observed (I²=0%) (Figure 15) [14-16]. Given the borderline statistical significance and the limited number of included studies, this finding should be interpreted cautiously and does not provide strong evidence of a difference in postoperative stereopsis between the two procedures.

**Figure 15:**
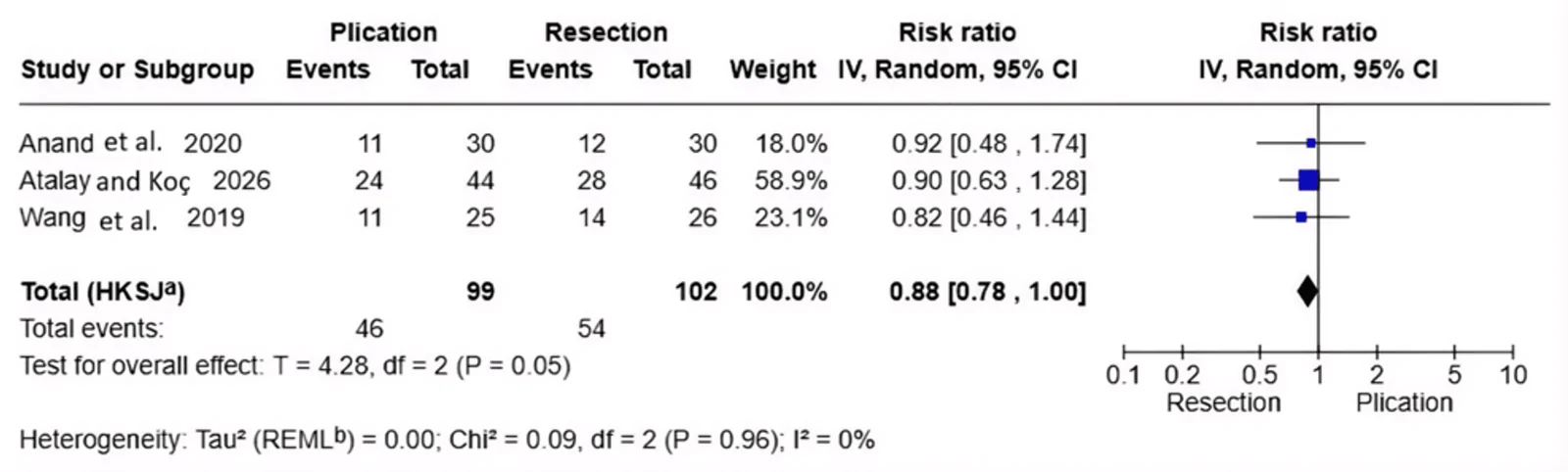
Forest plot comparing stereopsis outcomes between MR plication and resection. The pooled estimate shows no significant difference, with no heterogeneity. ^a^Confidence interval calculated using the Hartung-Knapp-Sidik-Jonkman (HKSJ) method. ^b^Tau² calculated using the restricted maximum-likelihood (REML) method. MR: medial rectus; REML: restricted maximum likelihood; HKSJ: Hartung-Knapp-Sidik-Jonkman; IV: inverse variance

Certainty of Evidence and Methodological Considerations

Using the GRADE approach, the certainty of evidence across outcomes ranged from moderate to very low. Surgical success and stereopsis were rated as low-certainty evidence, primarily because of concerns regarding risk of bias and imprecision. Postoperative deviation change, postoperative exodrift, and operative time were rated as very low-certainty evidence because of additional concerns related to inconsistency and substantial heterogeneity. The evidence for surgical dose-response was rated as moderate certainty, with downgrading due to risk of bias alone (Table 3).

**Table 3:** GRADE summary of findings. GRADE: Grading of Recommendations Assessment, Development and Evaluation; MD: mean difference

Outcome	Studies (design)	Participants	Effect estimate	Risk of bias	Inconsistency	Indirectness	Imprecision	Publication bias	Certainty
Surgical success rate	5 RCTs	268	RR: 0.86 (0.66 to 1.14)	Serious	Not serious	Not serious	Serious	Undetected	Low
Postoperative deviation change (PD)	6 RCTs	348	MD: 0.84 (-2.51 to 4.20)	Serious	Serious	Not serious	Serious	Undetected	Very low
Postoperative drift (exodrift)	4 RCTs	281	MD: 1.40 (-3.03 to 5.82)	Serious	Very serious	Not serious	Serious	Undetected	Very low
Surgical time/operative time	3 RCTs	155	MD: -2.07 min (-7.19 to 3.04)	Serious	Very serious	Not serious	Serious	Undetected	Very low
Dose-response outcome	3 RCTs	190	MD: -0.29 PD/mm (-0.48 to -0.09)	Serious	Not serious	Not serious	Not serious	Undetected	Moderate
Stereopsis	3 RCTs	201	RR: 0.88 (0.78 to 1.0)	Serious	Not serious	Not serious	Serious	Undetected	Low

Discussion

To our knowledge, this review may represent the first meta-analysis comparing medial rectus plication with medial rectus resection for exotropia that is restricted exclusively to randomized controlled trials. The comprehensive evaluation, encompassing six studies and 352 patients, found no statistically significant differences between the two procedures across the evaluated outcomes, despite including a spectrum of exotropia types and deviation magnitudes.

Our analysis found no statistically significant differences between the two techniques for the primary outcome of postoperative deviation change, indicating similar efficacy in achieving ocular alignment. Likewise, no statistically significant difference was observed in surgical success rates. Subgroup analyses were exploratory and generally showed no consistent differences between procedures. Furthermore, secondary outcomes such as postoperative exodrift, operative time, surgical dose-response, and stereopsis also exhibited no significant disparities between plication and resection. Overall, current short-term evidence suggests that medial rectus plication is a reasonable alternative to traditional resection; however, these findings should be interpreted in light of the methodological limitations, low to very-low certainty of evidence for most outcomes, and the relatively short follow-up periods of the included studies.

Interpretation in the Context of Previous Studies

The results of the present meta-analysis are consistent with the existing literature comparing plication and resection techniques. Wright and Lanier originally described rectus muscle plication as a modification of the rectus muscle tuck and as a muscle-tightening technique that avoids muscle disinsertion. Previous clinical studies have suggested that plication may produce a mechanical strengthening effect comparable to that achieved with resection [5,15].

Several comparative studies have reported similar surgical success rates and alignment outcomes between plication and resection procedures. For example, randomized trials in intermittent exotropia demonstrated comparable success rates and similar postoperative deviation change between the two techniques [7,18]. Likewise, Chaudhuri and Demer demonstrated that the surgical dose-effect of medial rectus plication was comparable to that of resection, supporting the biomechanical rationale of these procedures [3]. In contrast, Lee and Kim reported higher surgical success rates following medial rectus resection than plication in intermittent exotropia; however, their study was retrospective, included only one exotropia subtype, and had a longer follow-up period (two years), which may partly explain the difference from the present findings [19].

However, some observational studies have suggested that resection may provide slightly higher success rates or improved long-term stability [7]. These differences have been attributed to stronger scleral fixation and scar formation associated with muscle resection, which may theoretically result in greater mechanical stability compared with plication. Nevertheless, other studies have demonstrated similar postoperative drift patterns between the two techniques, indicating that long-term alignment stability may be comparable [4]. The findings of the present analysis support the latter interpretation. When pooled across randomized trials, no statistically significant difference between the two procedures was observed for the evaluated short-term outcomes.

Advantages of the Present Meta-Analysis Compared With Previous Reviews

Our meta-analysis offers several methodological improvements over prior reviews [20]. First, it included only RCTs, improving evidence quality and minimizing bias. Second, it expanded the scope to a broader range of clinically relevant outcomes (e.g., exodrift, operative time, surgical dose-response, and stereopsis), providing a more comprehensive evaluation. Third, it incorporated recent randomized studies, offering an updated synthesis of evidence. Fourth, it performed systematic subgroup and sensitivity analyses to explore heterogeneity. Finally, the inclusion of a GRADE assessment transparently evaluates the certainty of evidence, improving clinical interpretability.

Clinical Implications

From a clinical perspective, the current short-term evidence suggests that medial rectus (MR) plication may be a reasonable alternative to MR resection for the surgical management of exotropia, as no statistically significant differences were observed in postoperative deviation change or surgical success between the two procedures.

MR plication offers several theoretical advantages. It preserves the anterior ciliary vessels, which may reduce the risk of anterior segment ischemia during multiple muscle surgeries [4]. It is also potentially reversible in the early postoperative period, avoids the risk of slipped or lost muscles, and may be technically simpler. While differences in operative time and intraoperative trauma have not been consistently demonstrated in pooled analyses, the vessel-sparing nature of plication may make it an attractive option for patients undergoing multiple or repeat rectus muscle procedures [3]. Emerging evidence also suggests that preserving the distal myotendinous region during plication may maintain ocular proprioceptive mechanisms, potentially contributing to postoperative motor control; however, these findings remain preliminary and require confirmation in future comparative trials [21].

Limitations

Despite several important strengths, this study also has limitations. The overall number of included trials and total sample size were relatively small, which may limit the statistical power of some analyses. Differences in surgical techniques, patient populations, and follow-up durations across studies may also contribute to heterogeneity. In addition, some subgroup analyses were based on a limited number of studies, which restricts the strength of conclusions regarding specific patient subgroups.

Future research directions

Future research should prioritize large multicenter randomized controlled trials with longer follow-up durations to comprehensively assess the long-term stability of surgical outcomes for both plication and resection procedures. Key areas for improvement include standardizing surgical dosing protocols and outcome definitions to enhance comparability across studies. Additionally, investigations should expand beyond motor alignment to explore functional outcomes, such as binocular vision recovery and patient-reported quality-of-life measures. Further biomechanical studies on extraocular muscle proprioception and vascular preservation are also recommended to identify patient populations that might benefit more from one surgical technique than the other.

## Conclusions

Medial rectus plication and medial rectus resection in the management of exotropia remain under investigation, as no statistically significant difference was found between the two procedures. Across various outcomes, including postoperative deviation change, surgical success, postoperative exodrift, operative time, and stereopsis, no consistent clinically meaningful differences were identified between the two procedures. While current evidence does not support the clear clinical superiority of either technique, plication offers theoretical procedural and physiological advantages. These include preserving anterior segment circulation and maintaining distal myotendinous proprioceptive structures, which may affect postoperative motor control. However, the available evidence is limited by methodological shortcomings, low to very-low certainty for most outcomes, and short follow-up durations (three to 12 months). Therefore, further high-quality randomized controlled trials with longer follow-up are needed to confirm these findings and determine whether specific patient subgroups might benefit more from one technique than the other.

## Appendices

**Table 4:** Search strategy and results for each database.

Database	Search strategy
PubMed	((esotropia[Title/Abstract]) OR (esotrop*[Title/Abstract]) OR (exotropia[Title/Abstract]) OR (exotrop*[Title/Abstract]) OR (strabismus[Title/Abstract]) OR (strabism*[Title/Abstract]) OR (convergent strabismus[Title/Abstract]) OR (convergen*[Title/Abstract]) OR (divergent strabismus[Title/Abstract]) OR (divergen*[Title/Abstract]) OR (internal strabismus[Title/Abstract]) OR (external strabismus[Title/Abstract]) OR (exodeviation[Title/Abstract]) OR (divergent squint[Title/Abstract])) AND ((resection[Title/Abstract]) OR (plication[Title/Abstract]) OR (tucking[Title/Abstract]) OR (symmetry surgery[Title/Abstract]) OR (asymmetry surgery[Title/Abstract]) OR (selective surgery[Title/Abstract]) OR (bilateral plication[Title/Abstract]) OR (bilateral resection[Title/Abstract]) OR (unilateral plication[Title/Abstract]) OR (unilateral resection[Title/Abstract]) OR (recess-resect[Title/Abstract]) OR (recess-plication[Title/Abstract]))
Scopus	( ( ( ( esotropia[Title / Abstract] ) OR ( esotrop*[Title / Abstract] ) OR ( exotropia[Title / Abstract] ) OR ( exotrop*[Title / Abstract] ) OR ( strabismus[Title / Abstract] ) OR ( strabism*[Title / Abstract] ) OR ( convergent strabismus[Title / Abstract] ) OR ( convergen*[Title / Abstract] ) OR ( divergent strabismus[Title / Abstract] ) OR ( divergen*[Title / Abstract] ) OR ( internal strabismus[Title / Abstract] ) OR ( external strabismus[Title / Abstract] ) OR ( exodeviation[Title / Abstract] ) OR ( divergent squint[Title / Abstract] ) ) ) AND ( ( ( resection[Title / Abstract] ) OR ( plication[Title / Abstract] ) OR ( tucking[Title / Abstract] ) OR ( symmetry surgery[Title / Abstract] ) OR ( asymmetry surgery[Title / Abstract] ) OR ( selective surgery[Title / Abstract] ) OR ( bilateral plication[Title / Abstract] ) OR ( bilateral resection[Title / Abstract] ) OR ( unilateral plication[Title / Abstract] ) OR ( unilateral resection[Title / Abstract] ) OR ( recess-resect[Title / Abstract] ) OR ( recess-plication[Title / Abstract] ) ) ) )
Web of Science	(((TS=esotropia OR TS=esotrop* OR TS=exotropia OR TS=exotrop* OR TS=strabismus OR TS=strabism* OR TS="convergent strabismus" OR TS=convergen* OR TS="divergent strabismus" OR TS=divergen* OR TS="internal strabismus" OR TS="external strabismus" OR TS=exodeviation OR TS="divergent squint")) AND (TS=resection OR TS=plication OR TS=tucking OR TS="symmetry surgery" OR TS="asymmetry surgery" OR TS="selective surgery" OR TS="bilateral plication" OR TS="bilateral resection" OR TS="unilateral plication" OR TS="unilateral resection" OR TS="recess-resect" OR TS="recess-plication"))
Cochrane Library	esotropia:ti,ab,kw OR esotrop*:ti,ab,kw OR exotropia:ti,ab,kw OR exotrop*:ti,ab,kw OR strabismus:ti,ab,kw OR strabism*:ti,ab,kw OR "convergent strabismus":ti,ab,kw OR convergen*:ti,ab,kw OR "divergent strabismus":ti,ab,kw OR divergen*:ti,ab,kw OR "internal strabismus":ti,ab,kw OR "external strabismus":ti,ab,kw OR exodeviation:ti,ab,kw OR "divergent squint":ti,ab,kw resection:ti,ab,kw OR plication:ti,ab,kw OR tucking:ti,ab,kw OR "symmetry surgery":ti,ab,kw OR "asymmetry surgery":ti,ab,kw OR "selective surgery":ti,ab,kw OR "bilateral plication":ti,ab,kw OR "bilateral resection":ti,ab,kw OR "unilateral plication":ti,ab,kw OR "unilateral resection":ti,ab,kw OR "recess-resect":ti,ab,kw OR "recess-plication":ti,ab,kw #1 AND #2 AND #3
Google Scholer	(esotropia OR esotrop* OR exotropia OR exotrop* OR strabismus OR strabism* OR “convergent strabismus” OR convergen* OR “divergent strabismus” OR divergen* OR “internal strabismus” OR “external strabismus” OR exodeviation OR divergent squint) AND ( resection OR plication OR tucking OR “symmetry surgery” OR “asymmetry surgery” OR “selective surgery” OR “bilateral plication” OR “bilateral resection” OR “unilateral plication” OR “unilateral resection” OR “recess-resect” OR “recess-plication”)
